# Changes in the Influence of Alcohol-Paired Stimuli on Alcohol Seeking across Extended Training

**DOI:** 10.3389/fpsyt.2016.00169

**Published:** 2016-10-10

**Authors:** Laura H. Corbit, Patricia H. Janak

**Affiliations:** ^1^School of Psychology, The University of Sydney, Sydney, NSW, Australia; ^2^Department of Psychological and Brain Sciences, Krieger School of Arts and Sciences, Johns Hopkins University, Baltimore, MD, USA; ^3^Department of Neuroscience, Johns Hopkins School of Medicine, Johns Hopkins University, Baltimore, MD, USA

**Keywords:** outcome devaluation, Pavlovian–instrumental transfer, ethanol, stimuli, habit learning

## Abstract

Previous work has demonstrated that goal-directed control of alcohol-seeking and other drug-related behaviors is reduced following extended self-administration and drug exposure. Here, we examined how the magnitude of stimulus influences on responding changes across similar training and drug exposure. Rats self-administered alcohol or sucrose for 2 or 8 weeks. Previous work has shown that 8 weeks, but not 2 weeks of self-administration produces habitual alcohol seeking. Next, all animals received equivalent Pavlovian conditioning sessions where a discrete stimulus predicted the delivery of alcohol or sucrose. Finally, the impact of the stimuli on ongoing instrumental responding was examined in a Pavlovian–instrumental transfer (PIT) test. While a significant PIT effect was observed following 2 weeks of either alcohol or sucrose self-administration, the magnitude of this effect was greater following 8 weeks of training. The specificity of the PIT effect appeared unchanged by extended training. While it is well established that evaluation of the outcome of responding contributes less to behavioral control following extended training and/or drug exposure, our data indicate that reward–predictive stimuli have a stronger contribution to responding after extended training. Together, these findings provide insight into the factors that control behavior after extended drug use, which will be important for developing effective methods for controlling and ideally reducing these behaviors.

## Introduction

While early recreational drug use is largely driven by the reinforcing properties of the drug, over extended use, many of the positively reinforcing effects of drugs are diminished. The continued drug use by some individuals under such conditions suggests that drug-seeking behavior has become disconnected from expectations regarding the outcome of that behavior. An increasing automatization of responding could explain this shift. Although the notion that responding for drug rewards becomes habitual is prevalent in the addiction field (1–3), it has only been relatively recently that empirical studies have directly assessed this claim. There is now accumulating evidence that with prolonged drug use, control of drug-seeking behaviors transitions from flexible and goal-directed to habitual.

Tests developed in the animal learning field can dissociate goal-directed actions from response habits. Goal-directed actions rely on their relationship to, and the value of, their associated outcome. Thus, responding tracks both the action–outcome contingency and current value of the outcome and is normally reduced when either the former is degraded or the latter reduced (4, 5). In contrast to the knowledge of the action–outcome relationship and evaluation of outcome value that characterize goal-directed behaviors, habits are argued to rely on an independent learning process. Habits are acquired as stimulus–response (S–R) associations that are gradually strengthened each time a response is reinforced, explaining why the relative dominance of habitual control grows with extended training (6, 7). Because habitual responding is controlled by an S–R association that does not include a representation of the outcome or its value, changes in the value of the outcome have no immediate effect on the performance of habitual responses (6, 8). Thus, by specifically manipulating outcome value or the action–outcome contingency and observing consequent effects on performance, the outcome devaluation and contingency degradation tests have become useful tools for identifying goal-directed and habitual actions (5), and evidence of drug-induced habits has largely been derived from studies using these tests. The outcome devaluation task, in particular, has been effective in demonstrating that drug exposure can promote habitual control. For example, sensitizing doses of psychostimulant drugs prior to training with food reward can promote rapid habit formation evidenced by impaired sensitivity to devaluation (9–13). Likely of more direct relevance to human addiction, extensive, but not limited self-administration training with cocaine (14), alcohol (15), or nicotine (16) results in drug seeking that is no longer sensitive to outcome devaluation.

These failures of goal-directed control imply that drug seeking is habitual; nonetheless, they do not directly assess the S–R learning that is thought to underlie habitual behavior. While habits are thought to rely on the formation of an S–R association, the stimuli that support the S–R association and consequently, habitual performance in a free operant paradigm are typically poorly defined. The S–R association is established during instrumental training when the response is repeatedly reinforced, incrementally strengthening the association between that response and situational cues that are present. These stimuli could be derived from the physical context. However, since these cues are incidental, it is not clear what exact information the animal uses (context, elements of the context, sight of the lever, aspects of their own behavior, the outcome itself, etc.), and this could differ animal-by-animal, making the stimuli difficult to manipulate. While there is an independent literature implicating drug-related stimuli in craving and subsequent relapse risk (17–21), how the nature of such influences changes across the course of extended drug use has rarely been assessed and deserves further study, particularly in relation to whether behavior is under goal-directed or habitual control.

Stimulus influences in general can be readily manipulated and examined using the Pavlovian–instrumental transfer (PIT) task. This task examines the influence of stimuli on the choice and vigor of responses that earn drug or other rewards. It involves three independent stages. In the Pavlovian conditioning phase, a stimulus or stimuli are paired with an outcome or outcomes (such as drug, food, or other reward). Separately, animals are trained to perform one or more instrumental actions, such as a lever-press response, to earn reward. Importantly, the Pavlovian stimuli are not present during the instrumental training phase. In the final test stage, the instrumental action(s) is available and, for the first time, the Pavlovian stimuli are presented in order to assess their influence on instrumental performance. Changes in instrumental responding in the presence of the Pavlovian stimuli relative to stimulus-free periods constitute the PIT effect. Tests of PIT are typically conduced under extinction conditions (i.e., no rewards are delivered following either stimulus presentations or performance of the instrumental response) to prevent new learning at the time of testing and to allow confidence that effects rely on associations previously established during training. There is some evidence that the magnitude of PIT effects increases with extended instrumental training with food reward (7); however, the relationship between the amount of training and the magnitude of PIT effects is not straightforward (22). Furthermore, how stimulus effects related to drug seeking may change over the course of extended training has not been extensively investigated.

We have previously shown that an alcohol-seeking response is sensitive to devaluation of the alcohol reward following 2 weeks, but not 8 weeks of training, providing evidence of a failure of goal-directed control after this extended training and drug exposure (11, 12, 15). In the current study, we examined the influence of alcohol-predictive stimuli on an alcohol-seeking response across this same timeframe. Given that habits are thought to be driven by stimuli rather than outcome and that the relative dominance of the habit system increases across extended training, we predicted that stimulus influences on responding should increase with training, that is, the magnitude of the PIT effect would increase from 2 weeks of training, where behavior is goal-directed, to 8 weeks of training, where behavior is habitual. We compared any changes in the magnitude of the PIT effect in animals trained to self-administer alcohol versus sucrose reward. Furthermore, we tested whether the specificity of PIT changes over extended training.

## Materials and Methods

### Experiment 1: Pavlovian–Instrumental Transfer Following 2 or 8 Weeks of Alcohol Self-Administration

#### Subjects and Apparatus

Sixteen male Long–Evans rats (approximately 300 g at the start of the experiment; Harlan, Indianapolis, IN, USA) were singly housed with free access to food and water. This study was conducted in accordance with the recommendations of the National Institutes of Health Office of Laboratory Animal Welfare. All procedures were approved by the Institutional Animal Care and Use Committee of the Ernest Gallo Clinic and Research Center at the University of California, San Francisco, CA, USA. Training and testing took place in 16 Med Associates (East Fairfield, VT, USA) operant chambers housed within sound-attenuating shells. Each chamber was equipped with a pump fitted with a syringe that delivered a fixed volume of solution into a recessed magazine in the chamber when activated. The chambers contained retractable levers to the left and right of the magazine. A houselight mounted on the top-center of the opposite wall provided illumination.

#### Alcohol Acclimation in the Home Cage

To familiarize the rats with the taste and pharmacological effects of alcohol, they were given free access to 10% ethanol (10E) (v/v) in filtered water in the home cage, for 24 h/day for 14 days, followed by 14 days of 1-h access to 10E at the time that training would subsequently occur. Water was always available in a separate bottle fixed to the home cage. Rats were weighed daily, and EtOH consumption was recorded.

#### Instrumental Training

Animals were assigned to either a 2-week or an 8-week group (*N* = 8/group) in an effort to match home cage alcohol consumption. The 2-week group completed 14 daily training sessions, whereas the 8-week group completed 56 daily sessions before Pavlovian training and PIT testing. Training started with a single 30-min magazine training session, where 10E was delivered under a random time (RT) 60-s schedule. Rats were next trained to make a lever-press response to deliver small aliquots (0.1 ml) of 10E in 60-min sessions. The first 2 days of training were under a continuous reinforcement schedule; reinforcement was then shifted to a random ratio-2 schedule for 3 days, followed by a random ratio-3 schedule for the remainder of training. Animals failing to respond at levels sufficient to achieve alcohol intake of at least 0.3 g/kg for 5 out of 7 days/week were excluded from the study. In sum, for the experiments reported here, four animals were excluded on this basis; however, group sizes reported here reflect animals that met the instrumental training criterion as only those animals went on to Pavlovian conditioning and PIT testing. The reward receptacle was examined at the end of each session to ensure that the earned rewards were consumed; apart from the initial training day, this was always the case. At the end of instrumental training, animals were tested for sensitivity to outcome devaluation by outcome-specific satiety. These procedures and data are reported elsewhere (15).

#### Pavlovian Training

Pavlovian training and PIT testing followed our previous published methods (23). Briefly, following instrumental training and devaluation testing, the rats received eight sessions of Pavlovian conditioning. Two auditory stimuli (white noise and clicker) served as conditional stimuli. One of these stimuli (CS+) was paired with ethanol delivery, while the other stimulus (CS−) had no programed consequences (counterbalanced). Six presentations of each stimulus were given in each session in random order separated by periods in which no stimuli were present. The average length of the intertrial interval varied but on average was 4.5 min. The stimulus presentations were 2-min long. During each CS+ presentation, 0.2 ml of 10E was delivered on a RT 30-s schedule. Because the schedule of 10E delivery was random, the number of outcomes varied across sessions. On average, the animals received 4.8 ml of 10E across the 75-min session, which should lead to significant blood alcohol levels. The number of magazine entries during each stimulus and pre-stimulus interval of equal length (2 min) was measured. The magazine was inspected at the end of the training sessions to ensure that the solutions had been consumed.

#### Pavlovian–Instrumental Transfer Test

Rats received a single PIT test in which the lever was available, and each stimulus was presented twice interspersed with intervals of no stimulus (Ø). No rewards were delivered during testing. The 22-min test contained eight, 2 min bins [two white noise trials (N) and two clicker trials (C) alternated with four Ø trials in the following order: N, C, C, N]. Each stimulus presentation was separated from the subsequent baseline (Ø) interval by 1 min, and there was an additional 2-min extinction period prior to the first pre-CS interval.

#### Data Analysis

Data were analyzed using analysis of variance (ANOVA). Significant main effects and interactions were analyzed with further ANOVA, and significant simple effects were examined with pairwise comparisons.

### Experiment 2: Pavlovian–Instrumental Transfer Following 2 or 8 Weeks of Sucrose Self-Administration

#### Subjects and Apparatus

The housing conditions and training apparatus were identical to those described in Experiment 1. Seventeen rats were assigned to either a 2-week (*N* = 8) or 8-week (*N* = 9) group. Rats were given free access to a 2% sucrose solution (2S) (weight/volume in filtered water) in the home cage for 48 h before training. The 2S solution was chosen based on pilot studies suggesting it would produce similar response rates as 10E.

#### Instrumental and Pavlovian Training and PIT Test

The training and test parameters were identical to those described for Experiment 1, except that 2S instead of 10E was used as the reinforcer.

### Experiment 3: The Specificity of Pavlovian–Instrumental Transfer Following 2 or 8 Weeks of Alcohol Self-Administration

#### Subjects and Apparatus

The housing conditions and training apparatus were identical to those described in Experiment 1.

#### Instrumental Training

Thirty rats were assigned to either a 2-week (*N* = 14) or 8-week (*N* = 16) group and trained to self-administer 10E as in Experiment 1.

#### Pavlovian Training

The rats received eight sessions of Pavlovian conditioning similar to that described above, except that two rewards (10E and 2S) were paired with the two stimuli (white noise and clicker). Six presentations of each stimulus were given in each session in random order separated by stimulus-free intervals. During each stimulus presentation, 0.2 ml of the appropriate solution was delivered on a RT 30-s schedule. All other parameters matched those described in Experiment 1.

#### Pavlovian–Instrumental Transfer

The PIT test was identical to that described in Experiment 1.

## Results

### Experiment 1: Pavlovian–Instrumental Transfer Is Enhanced Following Extended Alcohol Self-Administration

#### Training

**Figure 1:**
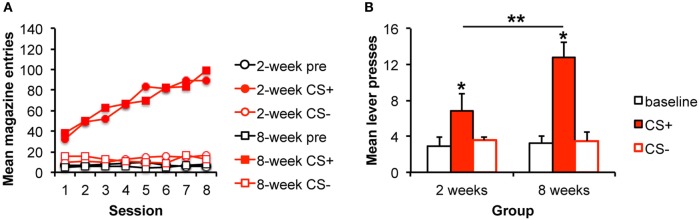
**Pavlovian–instrumental transfer is greater following extended alcohol self-administration**. **(A)** Mean magazine entries (+SEM) during the pre-CS (baseline) period and presentations of the CS+ and CS− across days of Pavlovian training for the 2- and 8-week training groups. **(B)** Mean lever presses (+SEM) during the pre-CS (baseline) period and presentations of the CS+ and CS− during the Pavlovian–instrumental transfer test. The excitatory effects of the CS+ are greater for the 8-week group. *indicates responding during the CS+ is greater than during the baseline period, *p* < 0.05. **indicates responding is greater for the 8-week group than for the 2-week group, *p* < 0.05.

Response rates at the end of instrumental training for the 2- and 8-week groups are included in Table 1. Magazine entries across days of Pavlovian training are shown in Figure 1A. Responding during the CS+ increased across days relative to responding during either the CS− or the baseline period. ANOVA confirmed these observations with a significant effect of stimulus [*F*(2,28) = 82.4, *p* < 0.001], day [*F*(7,98) = 12.3, *p* < 0.001], and an interaction between these factors [*F*(14,196) = 20.1, *p* < 0.001]. Importantly, there was no effect of group [*F*(1,14) = 0.002, *p* = 0.961], and none of the interactions involving group were significant (*F*s < 1).

**Table 1:** **Instrumental response rates for alcohol prior to PIT testing in Experiment 1**.

Group	Lever presses	Earned alcohol	g/kg ethanol
2-week	94.2 (11.3)	2.9 (0.37) ml	0.55 (0.07)
8-week	83.1 (10.9)	3.1 (0.39) ml	0.45 (0.06)

*Mean (SEM) lever-press responses, volume of alcohol consumed, and gram/kilogram ethanol levels for the final 3 days of instrumental training*.

#### Pavlovian–Instrumental Transfer

We tested the hypothesis that stimulus influences on responding would grow with extended training by testing the magnitude of the PIT effect following 2 or 8 weeks of training. The data are presented in Figure 1B, which shows that the alcohol-predictive stimulus elevated the alcohol-seeking response from baseline, and that this effect was bigger after 8 weeks of training. The analyses confirmed these impressions revealing an effect of stimulus [pre, CS+, CS−; *F*(2,28) = 16.7, *p* < 0.001], no effect of group [*F*(1,14) = 1.4, *p* = 0.253], but an interaction between these factors [*F*(2,28) = 4.3, *p* = 0.024]. To examine the nature of the interaction and to address whether the impact of the CS+ was specifically enhanced with extended training, simple effects analyses comparing groups for each level of stimulus were conducted. The groups did not differ in responding during the baseline [pre; *F*(1,15) = 0.45, *p* = 0.511] or CS− [*F*(1,15) = 0.51, *p* = 0.486] intervals. However, responding during the CS+ was greater for the 8-week than for the 2-week group [*F*(1,15) = 5.21, 0.039]. Furthermore, responding during the CS+ was greater than during the baseline period for both the 2- and 8-week groups [2 weeks: *F*(1,7) = 2.5, *p* = 0.041; 8 weeks: *F*(1,7) = 6.31, *p* < 0.001] confirming significant PIT in each group.

### Experiment 2: Pavlovian–Instrumental Transfer Following 2 or 8 Weeks of Sucrose Self-Administration

#### Training

**Figure 2:**
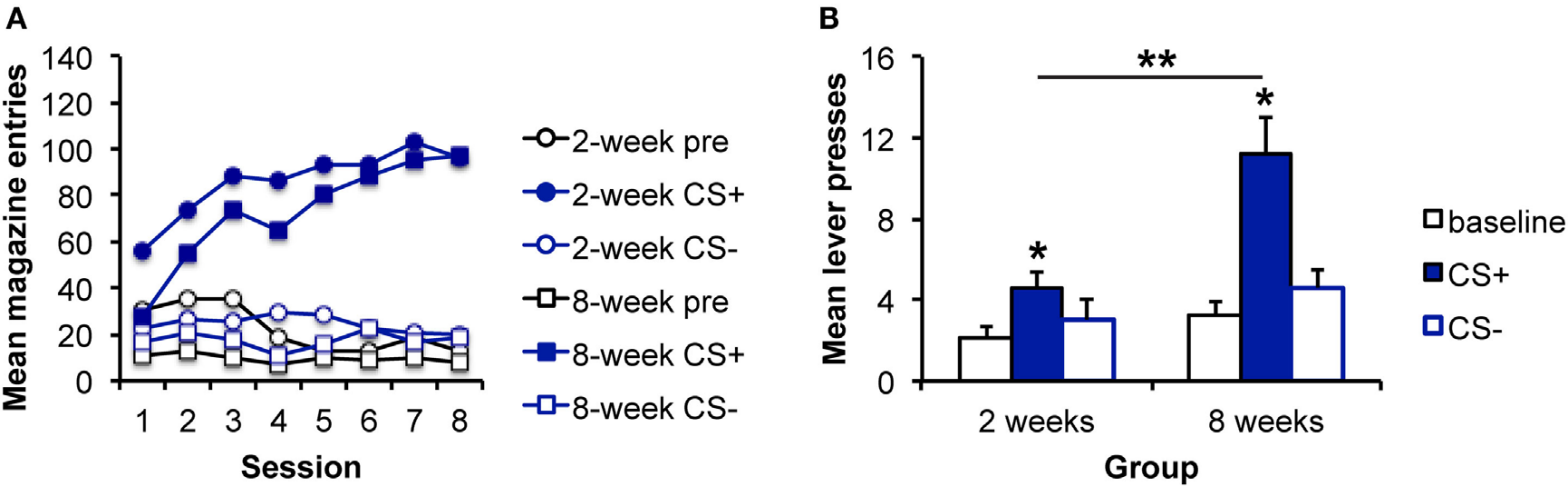
**Pavlovian–instrumental transfer is greater following extended sucrose self-administration**. **(A)** Mean magazine entries (+SEM) during the pre-CS (baseline) period and presentations of the CS+ and CS− across days of Pavlovian training for the 2- and 8-week training groups. **(B)** Mean lever presses (+SEM) during the pre-CS (baseline) period and presentations of the CS+ and CS− during the Pavlovian–instrumental transfer test. The excitatory effects of the CS+ are greater for the 8-week group. *indicates responding during the CS+ is greater than during the baseline period, *p* < 0.05. **indicates responding is greater for the 8-week group than for the 2-week group, *p* < 0.05.

Instrumental response rates at the end of training are shown in Table 2. Pavlovian training is shown in Figure 2A. As with alcohol reward, responding during the CS+ increased across days relative to responding during either the CS− or baseline period. ANOVA confirmed these observations with a significant effect of stimulus [*F*(2,30) = 97.5, *p* < 0.001], day [*F*(7,105) = 3.0, *p* = 0.006], and an interaction between these factors [*F*(14,210) = 9.6, *p* < 0.001]. Again, there was no effect of group [*F*(1,15) = 3.0, *p* = 0.103], and none of the interactions involving group were significant (*F*s < 1).

**Table 2:** **Instrumental response rates for sucrose prior to PIT testing in Experiment 2**.

Group	Lever presses	Earned sucrose
2-week	68.6 (7.9)	2.9 (0.35) ml
8-week	94.4 (19.1)	3.3 (0.79) ml

*Mean (SEM) lever-press responses and volume of sucrose consumed for the final 3 days of instrumental training*.

#### Pavlovian–Instrumental Transfer

Data from the PIT test are shown in Figure 2B, which shows that a sucrose-predictive stimulus also elevates performance of a sucrose-seeking response, and that this effect appears to grow with extended training. Analyses revealed an effect of stimulus [*F*(2,30) = 8.64, *p* = 0.001], an effect of group [*F*(1,15) = 5.66, *p* = 0.029], and an interaction between these factors [*F*(2,30) = 4.61, *p* = 0.017]. As above, to address whether the impact of the CS+ was enhanced with extended training, simple effects analyses comparing groups for each level of stimulus were conducted. The groups did not differ in responding during the baseline [pre; *F*(1,16) = 0.06, *p* = 0.808] or CS− [*F*(1,16) = 0.76, *p* = 0.395] intervals. However, responding during the CS+ was greater for the 8-week, than for the 2-week group [*F*(1,16) = 8.2, 0.011]. Furthermore, responding during the CS+ was greater than during the baseline period for both the 2- and 8-week groups [2 weeks: *F*(1,7) = 8.59, *p* = 0.022; 8 weeks: *F*(1,8) = 16.58, *p* = 0.002] confirming significant PIT in each group.

### Experiment 3: The Specificity of Pavlovian–Instrumental Transfer Following 2 or 8 Weeks of Alcohol Self-Administration

#### Training

**Figure 3:**
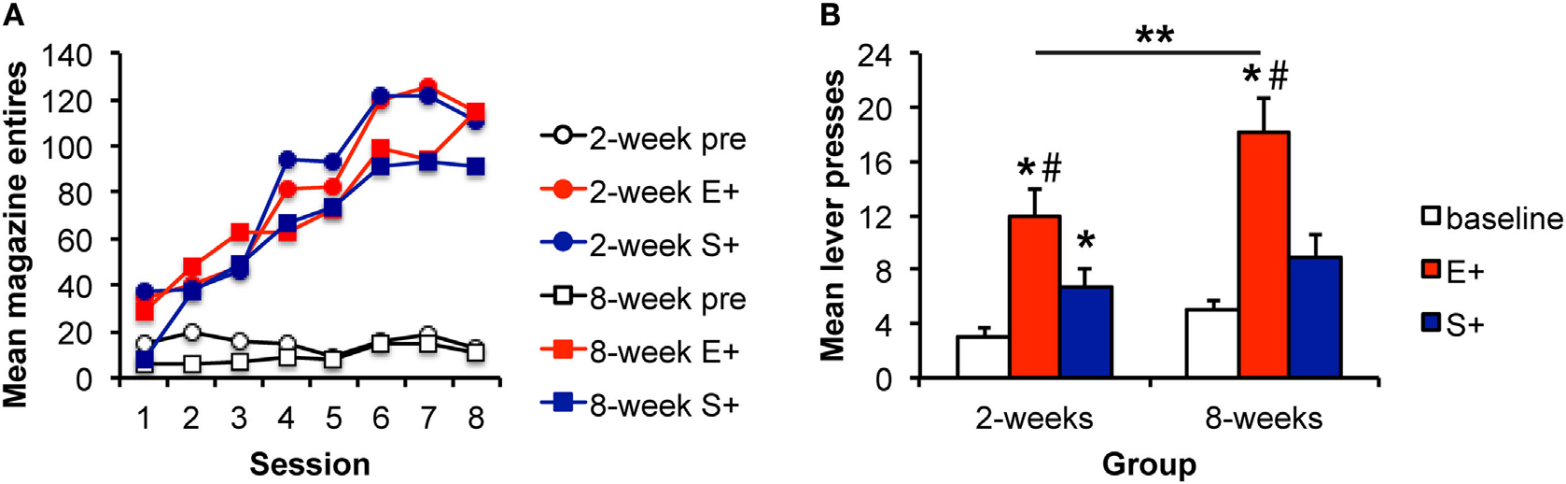
**The magnitude of the Pavlovian–instrumental transfer effect is greater following extended training, but the specificity is unchanged**. **(A)** Mean magazine entries (+SEM) during the pre-CS (baseline) period and presentations of the alcohol-predictive (E+) and sucrose-predictive (S+) stimuli across days of Pavlovian training for the 2- and 8-week training groups. **(B)** Mean lever presses (+SEM) during the pre-CS (baseline) period and presentations of the E+ and S+ during the Pavlovian–instrumental transfer test. The excitatory effects of the E+ are greater for the 8-week group. While S+ enhanced responding from baseline, this effect did not differ for the 2- and 8-week groups. *indicates responding during the CS+ is greater than during the baseline period, *p* < 0.05. **indicates responding is greater for the 8-week group than for the 2-week group, *p* < 0.05. ^#^indicates responding is greater during E+ than during S+, *p* < 0.05.

Instrumental response rates at the end of training are shown in Table 3. Pavlovian training is shown in Figure 3A. Responding during both stimuli increased similarly across days relative to responding during the baseline period. ANOVA confirmed these observations with a significant effect of stimulus [*F*(2,56) = 78.8, *p* < 0.001], day [*F*(7,196) = 47.8, *p* < 0.001], and an interaction between these factors [*F*(14,392) = 18.8, *p* < 0.001]. The stimulus effect was driven by increased responding during the stimuli relative to the baseline period. Responding during E+ and S+ did not differ [*F*(1,28) = 0.426, *p* = 0.519]. Again, there was no effect of group [*F*(1,28) = 1.6, *p* = 0.223], and none of the interactions involving group were significant (*F*s < 1).

**Table 3:** **Instrumental response rates for alcohol prior to PIT testing in Experiment 3**.

Group	Lever presses	Earned alcohol	g/kg
2-week	97.8 (14.4)	3.0 (0.39) ml	0.51 (0.09)
8-week	106.6 (8.9)	3.6 (0.38) ml	0.68 (0.07)

*Mean (SEM) lever-press responses, volume of alcohol consumed, and gram/kilogram ethanol levels for the final 3 days of instrumental training*.

#### Pavlovian–Instrumental Transfer

Data from the PIT test are shown in Figure 3B. There was an effect of stimulus [*F*(2,56) = 29.38, *p* < 0.001] and an effect of group [*F*(1,28) = 5.86, *p* = 0.023]. The interaction between these factors was not significant [*F*(2,56) = 2.39, *p* = 0.101] potentially because baseline responding was slightly higher in the 8-week group in this experiment. Based on the results of Experiments 1 and 2, we further explored whether the magnitude of the stimulus effects differed between groups. The groups did not differ in responding during the baseline [pre; *F*(1,29) = 3.23, *p* = 0.083]. However, responding during the E+ was greater for the 8-week, than for the 2-week group [*F*(1,29) = 5.82, 0.023]. Responding during the S+ did not differ between groups [*F*(1,29) = 0.77, *p* = 0.389]. Responding during the E+ was greater than during the baseline period for both the 2- and 8-week groups [2 weeks: *F*(1,13) = 35.25, *p* < 0.001; 8 weeks: *F*(1,14) = 32.79, *p* < 0.001]. Responding was also greater during the S+ for the 2-week group [*F*(1,13) = 8.16, *p* = 0.014] but failed to reach significance for the 8-week group [*F*(1,14) = 4.18, *p* = 0.059], overall confirming PIT effects in each group. Finally, responding during the E+ was greater than responding during the S+ in both the 2- and 8-week groups [F(1,13) = 6.36, *p* = 0.026; *F*(1,14) = 13.09, *p* = 0.003], providing evidence of specific PIT in addition to some general excitatory effects of the S+.

## Discussion

Previous work has shown that exposure to drugs, including alcohol, promotes the development of habitual control of responding. The extant data have largely been generated using the outcome devaluation task, where insensitivity to changes in the value of the outcome produced by responding demonstrates a lack of goal-directed control. Such findings are taken as evidence of habitual control since the outcome of responding is not part of the underlying associative structure that supports habitual behavior, and as such, manipulations of the outcome are expected to have no immediate effect on performance of a habitual response. Nonetheless, since habit learning is thought to rely on an independent learning process involving the formation of associations between stimuli present when responding is reinforced and the response itself, it is reasonable to expect changes in the influence of stimuli on responding as behavior transitions from goal-directed to habitual. While there is some evidence that stimulus influences grow with the development of habitual control (7), this has not been explored with drug reward, where drug-related stimuli are thought to contribute to sustained drug use and precipitate relapse following periods of abstinence.

Here, we find that the magnitude of the PIT effect is greater following 8 weeks of self-administration training than it is after 2 weeks, time points where related work has shown responding to be habitual and goal-directed, respectively, based on sensitivity to outcome devaluation (15). This effect is not explained by changes in overall response rates, which were similar for the 2- and 8-week training groups. Importantly, the amount of Pavlovian training was the same for the two groups, and the measure of Pavlovian performance during training, the magazine entry response, despite including both CS- and US-related responding, did not differ between groups. This suggests that it is not that the strength of the Pavlovian conditioning differs, but that with extended instrumental training, the susceptibility of the instrumental response to Pavlovian influences increases. Similar results were found in animals trained to self-administer alcohol or sucrose reward suggesting that this phenomenon relates to extended training rather than something specific about drug reward. Of note, the previous study by Holland (7), showing evidence of enhanced PIT with extended training, also used natural rewards; thus, the current finding with sucrose reward is not entirely unexpected. It is important to note that while few studies have manipulated the amount of training to examine effects on PIT within a single study, a meta-analysis performed by Holmes et al. (22) found a complex relationship between the amount of training and the magnitude of PIT effects. For example, they found that PIT effects were greater with more instrumental training when instrumental training was conducted after, but not before, the Pavlovian training phase in apparent contrast to the current findings. However, the meta-analysis only included studies that trained rats on interval schedules and excluded studies using drug, including alcohol reward. Further, the range of instrumental training for studies included in the analysis was 2–20 sessions with the majority using 6–12 sessions, which is a fairly narrow range. Rats in the current experiments underwent almost three times as much training as the maximum reported by Holmes et al. (22), reinforcement was according to ratio schedules, which could produce different learning and performance patterns than interval schedules, and, in Experiments 1 and 3, rats earned alcohol reward. With these important procedural details in mind, it is not clear that the results of the meta-analysis can be extended to the current results. Nonetheless, it appears that multiple factors contribute to the magnitude of PIT effects, and even the relationship with the amount of training may be complex, meaning enhanced PIT may not always be observed following extended training.

Interestingly, an experimental study included in Holmes et al. (22) found that extensive (16 sessions) Pavlovian training reduced rather than enhanced PIT in comparison to shorter training (4 sessions). They interpreted this result in terms of response competition as they also found evidence of increased magazine entries in the extensively trained group. However, absolute response rates for the lever-press and magazine entry responses were not high in relation to the 2-min stimulus interval, suggesting response competition is less likely, although an effect of unmeasured Pavlovian responses in addition to the magazine response can not be ruled out. In the current study, magazine entries during Pavlovian training that followed instrumental training did not differ between groups. Furthermore, for response competition to account for the current results, this competition would have to be greater in the 2-week groups. Further experimentation would be required to provide any support for such a claim; however, as it was the amount of instrumental rather than Pavlovian training that varied in the current study, it seems more likely that some change to the nature of instrumental performance between groups is responsible for the effects observed here.

As noted above, while habits are thought to rely on an S–R association, the stimuli that support habitual performance in a free operant paradigm are typically poorly defined. One possibility is that these stimuli are derived from the physical context. Indeed, there is some evidence that contextual stimuli can contribute to habitual responding. For example, studies using designs where goal-directed and habitual responses are generated in the same animal train these two responses in distinct contexts that differ in a range of visual and tactile properties (24, 25). Furthermore, instrumental performance is sometimes decreased when animals are tested in a context that is distinct to where they were trained, suggesting that the context contributes partially to instrumental performance (26). In contrast, the PIT procedure measures the effects of stimuli conditioned in a separate Pavlovian training phase rather than those that are incidentally present as animals perform the instrumental response. While it does not directly assess the strength of the S–R association thought to underlie response habits, it nonetheless provides evidence of the susceptibility of instrumental responding to Pavlovian influences. How the independently trained Pavlovian stimuli interact with the S–R association thought to underlie responding is currently unknown. In addition to a role for the training context, it is also possible that the animals’ own behavior sets the occasion for further responses or otherwise contributes to the S that drives S–R based responding. For example, animals may learn to follow magazine entry with a sequence of lever-press responses and as such, CS-elicited magazine entries could provoke additional lever presses in the presence of the CS. To the extent that behavior is more automatized following extended training or that sequences of behavior have been organized into “chunks,” it is possible that such effects could grow with extended training and account for the elevated PIT observed in the 8-week groups. Future work involving detailed analyses of response microstructure within PIT testing could address these possibilities.

Another possibility is that the outcome serves not only as a reinforcer but also as a stimulus that directs subsequent responses. Strong evidence that animals use the outcome in this way comes from some elegant experiments by Ostlund and Balleine examining outcome-specific reinstatement effects (27). For example, they trained animals under circumstances where different outcomes (O1 and O2) not only served as reinforcers for responding (R1–O1; R2–O2) but also served as antecedents of the response. The critical manipulation was that the outcome of responding and that which preceded the subsequent response was either congruent (O1–R1–O1; O2–R2–O2) or incongruent (O1–R2–O2; O2–R1–O1). Ostlund and Balleine then tested the ability of, say, O1 to reinstate extinguished responding. They found that presentation of O1 reinstated R1 in the group with congruent training; however, O1 reinstated R2 after incongruent training suggesting that the antecedent O–R association is responsible for reinstatement of instrumental responding. Thus, outcomes can serve as stimuli to direct responding. Applying this to an expectancy- or cueing-based explanation of PIT (28, 29), presentation of S will retrieve a representation of the outcome it was trained with, which in turn, through this O–R association, will promote performance of a response also associated with that O. In the current experiments, the free operant training of a single response is most similar to the congruent training of Balleine and Ostlund (27), and it would be expected that the earned outcome, say alcohol, serves not only as a reinforcer but also as a signal for performance of a response that earns alcohol. Presentation of the E+ then can invigorate performance of the alcohol response to generate the observed PIT effect. Based on the results of Balleine and Ostlund (27), one would expect this effect to be selective, which would explain why in Experiment 3, the effects of E+ but not S+ grow with extended training. To explain the enhanced PIT following extended training, this view assumes that the strength of the O–R association is incrementally strengthened with extended training much the same as is suggested for the more general S–R association proposed to underlie habit learning. With a stronger O–R association, retrieval of O as a signal for responding by S should have a greater effect on responding in the extended training group, which could account for the enhanced PIT that was observed in these groups. Importantly, Balleine and Ostlund (27) found that while the magnitude of outcome-specific reinstatement effects was reduced by devaluation, the specificity of these effects remained intact, indicating that the influence of the outcome on response selection does not depend on outcome value. This finding parallels reports that outcome-specific PIT is not dependent on outcome value and explains how PIT effects could grow under conditions where outcome value plays little role in controlling performance (that is, the devaluation-insensitive performance of the extended training groups).

Several different types of PIT have been identified. Stimuli may produce an enhancement (or suppression) of responding as a result of the motivational consequences of association with reinforcement generally (referred to as non-selective or general transfer). Alternatively, a stimulus may have quite specific effects impacting only response(s) associated with the same outcome as is predicted by the stimulus (referred to as specific transfer). As noted above, to explain such PIT effects, some theoretical accounts suggest that stimuli produce an expectancy regarding a particular outcome that, through a form of S–R process (S–O–R), elevates the performance of actions associated with the predicted outcome [e.g., Ref. (28, 30)]. Interestingly, when rats were trained with two stimuli that predicted alcohol and sucrose, respectively, while both stimuli elevated responding (on a response trained with alcohol), the alcohol-predictive stimulus was more effective in elevating responding, providing some evidence of specific PIT, and importantly, only the influence of the alcohol-predictive stimulus grew with extended training suggesting predominantly an outcome-specific effect rather than an energizing effect that should have impacted both stimuli. The meta-analysis conducted by Holmes et al. (22) found no evidence of changes to the specificity of PIT in experiments in designs that allowed examination of stimuli paired with the same or different outcomes as the target response. We have previously observed that alcohol-predictive stimuli are unique in that they also enhance performance of a response earning an alternate reward (sucrose) under training conditions that typically produce outcome-specific PIT (23). Thus, the lack of change in the influence of the sucrose stimulus on responding for alcohol in Experiment 3 is consistent with previous results (22). Whether the amount of training would have any impact on the previously reported general effects of an alcohol stimulus on responding for an alternate outcome, such as sucrose, was not tested in the current experiments and thus requires future experimentation.

While insensitivity to devaluation provides the most direct evidence of performance that is independent of goal value, it is worth noting several important demonstrations that the ability of stimuli to trigger responding does not depend on the predicted outcome being valuable at the time of testing. While the current study demonstrates particularly strong stimulus effects after training shown elsewhere to generate responding that is insensitive to devaluation, we did not examine the effects of devaluation on expression of PIT. However, others have shown that the ability of a stimulus to augment the performance of an action predicting the same outcome as the stimulus is not altered by outcome devaluation (31–33), although baseline response rates may be reduced. These types of findings demonstrate that the ability of stimuli to invigorate responding can be independent of evaluative processes related to the consequences of that responding. PIT effects also persist following manipulations that degrade the stimulus–outcome (S–O) contingency, such as extinction of S, pairing of S with a new outcome, or switching the S–O contingency to either a random or explicitly unpaired relationship with the outcome following initial training (34). These results, like those found with various recovery phenomena (spontaneous recovery, renewal, and reinstatement), suggest that S–O associations and their influence on behavior are persistent and difficult to change once established.

Of note, outcome devaluation and PIT tests are typically conducted under extinction conditions where reward is withheld, similar to other recovery phenomenon used to model human relapse. This differs from the human situation where drug seeking is likely to produce the desired drug. With this in mind, increases in the magnitude of effects, such as PIT, perhaps speak to the power of drug-associated stimuli to provoke the initiation of drug-seeking behaviors. The stronger these effects, the more likely stimuli are to trigger a drug-seeking response, which in real-world settings could result in drug use. Findings, such as the current results, suggest that the ability of stimuli to drive behavior increases under conditions that promote habitual control provide some insight into the factors that control responding when it is not generated by expectation and evaluation of a particular outcome, and it may help explain why habitual responding is resistant to change. Such findings may also improve understanding of the factors that contribute to relapse to drug use in individuals with a stated desire to abstain and who are aware of, but apparently insensitive to, the negative consequences of continued drug use.

## Author Contributions

LC conducted the experiments. LC and PJ designed the experiments, analyzed the data, and prepared the manuscript.

## Conflict of Interest Statement

The authors declare that the research was conducted in the absence of any commercial or financial relationships that could be construed as a potential conflict of interest.

## References

[1] Everitt BJ Robbins TW. Neural systems of reinforcement for drug addiction: from actions to habits to compulsion. Nat Neurosci (2005) 8(11):1481–9. 10.1038/nn1579 16251991

[2] Belin D Belin-Rauscent A Murray JE Everitt BJ. Addiction: failure of control over maladaptive incentive habits. Curr Opin Neurobiol (2013) 23(4):564–72. 10.1016/j.conb.2013.01.025 23452942

[3] Barker JM Taylor JR. Habitual alcohol seeking: modeling the transition from casual drinking to addiction. Neurosci Biobehav Rev (2014) 47:281–94. 10.1016/j.neubiorev.2014.08.012 25193245 PMC4258136

[4] Adams CD Dickinson A Instrumental responding following reinforcer devaluation. Quarterly J Exp Psychol B (1981) 33:109–21.

[5] Balleine BW Dickinson A. Goal-directed instrumental action: contingency and incentive learning and their cortical substrates. Neuropharmacology (1998) 37(4–5):407–19. 10.1016/S0028-3908(98)00033-1 9704982

[6] Adams CD Variations in the sensitivity of instrumental responding to reinforcer devaluation. Q J Exp Psychol (1982) 34(2):77–98. 10.1080/14640748208400878

[7] Holland PC. Relations between Pavlovian-instrumental transfer and reinforcer devaluation. J Exp Psychol Anim Behav Process (2004) 30:104–17. 10.1037/0097-7403.30.4.258 15078120

[8] Dickinson A Actions and habits: the development of behavioral autonomy. Philos Trans R Soc Lond B Biol Sci (1985) 308:67–78. 10.1098/rstb.1985.0010

[9] Nelson A Killcross S. Amphetamine exposure enhances habit formation. J Neurosci (2006) 26(14):3805–12. 10.1523/JNEUROSCI.4305-05.2006 16597734 PMC6674135

[10] Nordquist RE Voorn P de Mooij-van Malsen JG Joosten RN Pennartz CM Vanderschuren LJ. Augmented reinforcer value and accelerated habit formation after repeated amphetamine treatment. Eur Neuropsychopharmacol (2007) 17(8):532–40. 10.1016/j.euroneuro.2006.12.005 17275266

[11] Corbit LH Chieng BC Balleine BW. Effects of repeated cocaine exposure on habit learning and reversal by N-acetylcysteine. Neuropsychopharmacology (2014) 39(8):1893–901. 10.1038/npp.2014.37 24531561 PMC4059898

[12] Corbit LH Nie H Janak PH. Habitual responding for alcohol depends upon both AMPA and D2 receptor signaling in the dorsolateral striatum. Front Behav Neurosci (2014) 8:301. 10.3389/fnbeh.2014.00301 25228865 PMC4151333

[13] LeBlanc KH Maidment NT Ostlund SB Repeated cocaine exposure facilitates the expression of incentive motivation and induces habitual control in rats. PLoS One (2013) 8(4):e61355 10.1371/journal.pone.0061355 23646106 PMC3640016

[14] Zapata A Minney VL Shippenberg TS. Shift from goal-directed to habitual cocaine seeking after prolonged experience in rats. J Neurosci (2010) 30(46):15457–63. 10.1523/JNEUROSCI.4072-10.2010 21084602 PMC3073559

[15] Corbit LH Nie H Janak PH. Habitual alcohol seeking: time course and the contribution of subregions of the dorsal striatum. Biol Psychiatry (2012) 72:389–95. 10.1016/j.biopsych.2012.02.024 22440617 PMC3674580

[16] Clemens KJ Castino MR Cornish JL Goodchild AK Holmes NM. Behavioral and neural substrates of habit formation in rats intravenously self-administering nicotine. Neuropsychopharmacology (2014) 39(11):2584–93. 10.1038/npp.2014.111 24823947 PMC4207338

[17] Grusser SM Heinz A Raabe A Wessa M Podschus J Flor H. Stimulus-induced craving and startle potentiation in abstinent alcoholics and controls. Eur Psychiatry (2002) 17:188–93. 10.1016/S0924-9338(02)00666-1 12231263

[18] Le A Shaham Y Neurobiology of relapse to alcohol in rats. Pharmacol Ther (2002) 94:137–56. 10.1016/S0163-7258(02)00200-0 12191599

[19] Loeber S Croissant B Heinz A Mann K Flor H. Cue exposure in the treatment of alcohol dependence: effects on drinking outcome, craving and self-efficacy. Br J Clin Psychol (2006) 45:515–29. 10.1348/014466505X82586 17076961

[20] Fox HC Bergquist KL Hong KI Sinha R. Stress-induced and alcohol cue-induced craving in recently abstinent alcohol-dependent individuals. Alcohol Clin Exp Res (2007) 31:395–403. 10.1111/j.1530-0277.2006.00320.x 17295723

[21] Sinha R Fox HC Hong KA Bergquist K Bhagwagar Z Siedlarz KM. Enhanced negative emotion and alcohol craving, and altered physiological responses following stress and cue exposure in alcohol dependent individuals. Neuropsychopharmacology (2009) 34:1198–208. 10.1038/npp.2008.78 18563062 PMC2734452

[22] Holmes NM Marchand AR Coutureau E. Pavlovian to instrumental transfer: a neurobehavioural perspective. Neurosci Biobehav Rev (2010) 34(8):1277–95. 10.1016/j.neubiorev.2010.03.007 20385164

[23] Corbit LH Janak PH. Ethanol-associated cues produce general Pavlovian-instrumental transfer. Alcohol Clin Exp Res (2007) 31(5):766–74. 10.1111/j.1530-0277.2007.00359.x 17378919

[24] Killcross S Coutureau E. Coordination of actions and habits in the medial prefrontal cortex of rats. Cereb Cortex (2003) 13(4):400–8. 10.1093/cercor/13.4.400 12631569

[25] Gremel CM Costa RM. Orbitofrontal and striatal circuits dynamically encode the shift between goal-directed and habitual actions. Nat Commun (2013) 4:2264. 10.1038/ncomms3264 23921250 PMC4026062

[26] Thrailkill EA Bouton ME. Contextual control of instrumental actions and habits. J Exp Psychol Anim Learn Cogn (2015) 41(1):69. 10.1037/xan0000045 25706547 PMC4339261

[27] Balleine BW Ostlund SB Still at the choice-point. Ann N Y Acad Sci (2007) 1104(1):147–71. 10.1196/annals.1390.006 17360797

[28] Trapold MA Overmier JB The second learning process in instrumental learning. In: Black AA Prokasy WF, editors. Classical Conditioning II: Current Research and Theory. New York: Appleton-Century-Crofts (1972). p. 427–52.

[29] Corbit LH Balleine BW Learning and motivational processes contributing to Pavlovian-instrumental transfer and their neural bases: dopamine and beyond. In: Simpson EH Balsam PD, editors. Behavioral Neuroscience of Motivation. Switzerland: Springer International Publishing (2015). p. 259–89. 10.1007/7854_2015_388 26695169

[30] Corbit LH Balleine BW. Double dissociation of basolateral and central amygdala lesions on the general and outcome-specific forms of Pavlovian-instrumental transfer. J Neurosci (2005) 25(4):962–70. 10.1523/JNEUROSCI.4507-04.2005 15673677 PMC6725628

[31] Rescorla RA Transfer of instrumental control mediated by a devalued outcome. Anim Learn Behav (1994) 22(1):27–33. 10.3758/BF03199953

[32] Watson P Wiers RW Hommel B de Wit S Working for food you don’t desire. Cues interfere with goal-directed food-seeking. Appetite (2014) 79:139–48. 10.1016/j.appet.2014.04.005 24743030

[33] Colagiuri B Lovibond PF. How food cues can enhance and inhibit motivation to obtain and consume food. Appetite (2015) 84:79–87. 10.1016/j.appet.2014.09.023 25278431

[34] Delamater AR Effects of several extinction treatments upon the integrity of Pavlovian stimulus-outcome associations. Anim Learn Behav (1996) 24(4):437–49. 10.3758/BF03199015

